# Changes in the ideal body shape associated with adolescent rowing‐ergometry performance following a 6‐week training intervention: New scaling insights using three‐dimensional allometry

**DOI:** 10.1002/ejsc.12216

**Published:** 2024-11-02

**Authors:** Alan M. Nevill, Grace W. M. Walters, Karah J. Dring, Benjamin A. Nevill, Simon B. Cooper, John G. Morris

**Affiliations:** ^1^ Faculty of Education, Health and Wellbeing University of Wolverhampton Wolverhampton UK; ^2^ Department of Sport Science School of Science and Technology Sport, Health, and Performance Enhancement (SHAPE) Research Centre Nottingham Trent University Nottingham UK; ^3^ Princess Alexandra Hospital Harlow Essex UK

**Keywords:** 3‐D allometry, adolescents, allometric scaling, body size, rowing‐ergometry

## Abstract

Scaling, to remove the effects of body size, is an important methodological approach for enabling an equitable comparison of performance differences between individuals who vary in anthropometric characteristics. Many previous studies using scaling in sport have done so based on only one or two anthropometric characteristics, with only one study to date adopting a three‐dimensional approach. To apply a three‐dimensional allometric model to rowing ergometer performance (REP) in adolescents, and to detect whether key ‘scaling’ parameters remain stable when scaling REP both before and after a 6‐week training intervention. Novel three‐dimensional allometric models were used, incorporating body mass, stature and waist circumference (WC) to detect the most appropriate body size dimension(s) and scaling parameters associated with REP before and after a 6‐week training intervention. Using this more flexible and sensitive three‐dimensional allometry demonstrated that, following 6‐weeks of training, there was a change in the ideal body shape associated with REP. Before training, taller, but not heavier, adolescents performed better. After 6‐weeks of training, older participants with a greater body mass but smaller WC performed better. Scaling approaches are important for evaluating performance differences between individuals of differing body size. The findings from the current study (using a novel three‐dimensional allometry approach) emphasise that relatively subtle changes in individuals' behavioural characteristics, such as changes in their training/fitness status, can result in quite dramatic changes in the body dimension characteristics and scaling parameters deemed to be key for performance in activities such as REP.

## Highlights

- This study examined three‐dimensional allometric models of rowing ergometer performance (REP) before and after a 6‐week rowing training intervention.
- At baseline, stature was the most important determinant of REP.
- Following a 6‐week training intervention, the three‐dimensional allometric model revealed a positive effect of body mass and negative effect of waist circumference on REP.
- These findings highlight that allometric models are sensitive to changes over time, even in response to a relatively modest 6‐week training intervention.

## 1 INTRODUCTION

Physical proficiency in sports performance has been demonstrated to be strongly determined by body dimensions (Bongiovanni et al., 2021; Zhao et al., 2019). A common approach for examining the influence of different body dimensions on sporting performance and physical activities has been to use allometric scaling and modelling (Bustamante Valdivia et al., 2015; Giuriato et al., 2021; Lovecchio et al., 2019; Nevill, Holder, & Watts, 2009; Nevill, Tsiotra, et al., 2009; Silva et al., 2016; Watts et al., 2012). In paediatric sports science in particular, accounting for the influence of changes in body dimensions on physical performance as children and young people grow is both academically and practically essential (Welsman & Armstrong, 2021).

Allometric scaling has been applied to a number of physical performance measures in young people including motor performance (Bustamante Valdivia et al., 2015; Silva et al., 2016), performance on an array of fitness tests (Giuriato et al., 2021; Lovecchio et al., 2019; Nevill, Tsiotra, et al., 2009), sprinting (Watts et al., 2012) and football (Nevill, Holder, & Watts, 2009). However, these studies have only used the two dimensions of body mass and stature. Only using these two dimensions of body size however neglects the important component of body composition, which has also been shown to have an important influence on athletic performance (Knechtle et al., 2011; Slater et al., 2005). One recent study has introduced the novel consideration of including waist circumference (WC) (as a surrogate marker of body composition) in the allometric model for adolescents (Nevill et al., 2022). This investigation used a three‐dimensional allometry approach using height, body mass and WC to assess performance across six different athletic tests (including sprint speed, agility speed, endurance walking/running performance, upper limb strength, abdominal strength and lower limb strength). The results of this investigation highlighted that height and WC were consistently associated with all six performance tests, with more height always being beneficial and a bigger WC always being detrimental, but the contribution of body mass was dependant on whether the athletic test was weight bearing or not (Nevill et al., 2022). The results of this recent investigation therefore demonstrate the potential utility of a three‐dimensional allometry approach to provide novel insights into what constitutes the ‘ideal’ athletic shape associated with sports performance in children and adolescents (Nevill et al., 2022). However, to date, this is the only study to apply a three‐dimensional model which includes WC.

Given the relatively limited utilisation of three‐dimensional allometric modelling to date, the applicability of this approach for examining the influence of body dimensions on performance in various different sports and activities is unknown. For example, with regards to rowing performance, research demonstrates that body mass is an important determinant, whereby in young people heavier rowers out‐perform lighter rowers of the same level of skill (Nevill et al., 2010; Walters et al., 2023). Indeed, it has been reported that overweight/obese adolescents' row further on a maximal rowing test compared to healthy weight adolescents, both before and after 6‐weeks of rowing ergometer training (Walters et al., 2023). This research emphasises the importance of body mass as an important predictor of rowing‐ergometry performance. However, to date, three‐dimensional allometric modelling has not been applied to rowing performance; thus, the effects of other body dimensions beyond body mass remain unknown.

Furthermore, most research in this area of scaling sports performance variables to date has used a ‘static’ approach and only examined allometric modelling at a single point in time. It has thus been assumed that such a model will hold true over time, both as young people age and even in response to a period of exercise training. It is possible that the three‐dimensional models that best predict exercise performance may change over time, particularly where an anthropometric measure such as WC may change as a result of such training. However, this has not been examined to date.

Therefore, the aim of the present study was two‐fold: (i) to apply a three‐dimensional allometric approach to predict rowing performance in adolescents and (ii) to examine whether the three‐dimensional allometric model alters in response to a period of exercise training in adolescents.

## 2 METHODS

### 2.1 Study design and participants

Following approval from the institution's ethical advisory committee, adolescents (males and females, aged 12–13 years) from secondary schools in the Midlands area of the UK were invited to participate in a randomised control trial. Upon recruitment to the study, participants were randomly assigned to either an intervention group (6‐weeks of rowing ergometer training, twice per week during physical education lessons, consisting of 2 × 3 min bouts of high intensity rowing per session) or a control group (who continued with their usual habitual activities). In total, 102 participants completed the study (intervention group, *n* = 57; control group, *n* = 45). The results of the intervention itself have been reported elsewhere (Walters et al., 2023); this paper focuses on applying a three‐dimensional allometric scaling model to predict rowing ergometer performance (REP).

### 2.2 Anthropometric measures

At baseline and follow‐up, stature was measured using a portable stadiometer (Seca Leicester Height Measure; Cranlea) and body mass was measured using digital scales (Seca Electronic Scale 888; Cranlea). Furthermore, WC was measured using a tape measure (to the nearest 1 mm) at the narrowest point between the xiphoid process of the sternum and the iliac crest. WC was measured twice with the mean value used as the criterion measure. Should the first two measurements differ by >5%, a third measurement was taken and the median used as the criterion measure.

### 2.3 Rowing ergometer performance

REP was assessed via a 3 min all‐out maximal effort, as previously reported (Walters et al., 2023). A 3 min maximal test has been demonstrated to be a valid and reliable measure for assessing peak work output (Cheng et al., 2012). In brief, following a submaximal test that served as a warm‐up (3 × 3 min stages at 60, 71 and 86 W; interspersed by 90 s of rest), participants completed the maximal test. Participants were instructed to row as far as possible in the 3 min bout, with distance covered (in metres) used as the criterion measure. For consistency, the drag factor (resistance) (Concept2, 2023) was set at 105 during the maximal test.

### 2.4 Statistical analyses

In the past, a simple power function model, *Y* = *a* · *M* ^*b*^ · *ε*, has been used to identify the most appropriate body shape to normalize/scale physiological/performance variables (*Y*) for differences in body size, invariably adopting body mass (*M*) as the body size term (see Nevill et al., 1992). Recently however, a more flexible three‐dimensional allometric model incorporating mass (*M*), stature (*S*) and WC has been proposed to scale/identify the most appropriate body size dimension(s) associated with such performance variables (Nevill et al., 2022). It is these more flexible ‘three‐dimensional’ allometric models that we shall adopt to identify the most appropriate body dimension(s) associated with rowing‐ergometry performance (REP) both before and after a 6‐week training intervention, as follows:

(1) $$\text{REP}=a\cdot M^{k_{1}}\cdot S^{k_{2}}\cdot {\text{WC}}^{k_{3}}\cdot \exp\left(\text{age}\right)\cdot \varepsilon$$

where ‘*a*’ is the scaling constant and *k*_1_, *k*_2_ and *k*_3_ are scaling exponents for the body mass (*M*), stature (*S*) and WC respectively and *ε* is the multiplicative error ratio (Nevill et al., 1992). Training group and sex were incorporated into the model by allowing ‘*a*’ to vary for sex and training group (intervention vs. control group) as fixed factors. The model can be linearised with a log‐transformation, and multiple regression/ analysis of covariance (ANCOVA) can be used to estimate the body mass, height and WC exponents for REP having also controlled for age, training status and sex (Equation 2).

(2) $$\log\left(\text{REP}\right)=\log\left(a\right)+k_{1}\cdot \log\left(M\right)+k_{2}\cdot \log\left(S\right)+k_{3}\cdot \log\left(\text{WC}\right)+\text{age}+\log\left(\varepsilon \right)$$

In effect, log‐transformed REP becomes the dependent variable, with training group and sex incorporated as fixed factors, with log(*M*), log(*S*), log(WC) and age entered as the covariates. Having fitted the saturated model (all available predictor/body dimension variables described in Equation 1), an appropriate ‘parsimonious’ model can be obtained using ‘backward elimination’ (Draper & Smith, 1998) in which at each step the least important (non‐significant) body dimension variable is dropped from the current model. All analyses were performed in SPSS (version 29), with statistical significance accepted as *P* < 0.05.

## 3 RESULTS

### 3.1 Participant characteristics

One hundred and two participants were enrolled into the study and completed the 6‐week intervention, 57 in the training group (males *n* = 25; females *n* = 32) and 45 in the control group (males *n* = 12; females *n* = 33). Participant characteristics at baseline and follow‐up are provided in Table 1.

**TABLE 1:** Participant characteristics at baseline and follow‐up in the control and training groups.

	Control				Training				Mean difference in change (95% CI) (control vs. training)	*P* value
	*n* = 45				*n* = 57					
	Baseline	Follow‐up	Change	*P* value	Baseline	Follow‐up	Change	*P* value		
Sex (*n* male/female)	12/33				25/32					
Age (years)	12.63 ± 0.32				12.63 ± 0.33					
Stature (cm)	157.7 ± 7.3	158.2 ± 7.3	0.46 ± 1.07	0.003	157.0 ± 6.4	158.2 ± 6.6	1.26 ± 1.66	<0.001	0.8 (−1.37, −0.24)	0.003
Body mass (kg)	51.8 ± 12.7	51.7 ± 12.6	−0.06 ± 1.70	0.403	52.0 ± 9.5	52.3 ± 9.4	0.34 ± 1.27	0.049	0.4 (−0.99, 0.18)	0.088
Body mass index (kg · m^−2^)	20.7 ± 4.0	20.5 ± 4.0	−0.14 ± 0.69	0.092	21.0 ± 3.4	20.8 ± 3.2	−0.20 ± 0.62	0.018	0.06 (−0.20, 0.32)	0.318
Waist circumference (cm)	68.7 ± 8.7	69.2 ± 8.5	0.51 ± 2.30	0.073	69.4 ± 7.6	70.6 ± 7.8	1.18 ± 3.78	0.022	0.67 (−1.94, 0.60)	0.149
Maximal rowing test performance (m)	562.8 ± 54.9	553.2 ± 63.8	−9.51 ± 37.59	0.048	576.9 ± 63.9	596.7 ± 71.0	19.74 ± 59.78	0.016	29.25 (−49.51, −8.99)	0.003

*Note*: Data are mean ± standard deviation.

### 3.2 Pre‐training rowing ergometer performance

The simple power function model *Y* = *a* · *M* ^*b*^ · *ε* predicting the pre‐training REP identified a significant body mass exponent to be 0.151 (standard error [SE] = 0.055; *P* = 0.007, 95% Confidence Intervals [CI] 0.041–0.260; *R* ^2^ = 0.086 [Adjusted *R* ^2^ = 0.049]).

The ‘three dimensional’ allometric ANCOVA analysis of log‐transformed REP (pre‐training) identified no main effects of either training group, sex or a training‐by‐sex interaction (all *P* > 0.05). The ANCOVA analysis also revealed only one significant body size covariate, that being log‐transformed stature (Ln(*S*)), with an exponent of 0.769 (SE = 0.255; *P* = 0.003, 95% CI 0.263–1.276), although ‘age’ was close to significance (*P* = 0.062) (Table 2). Note that the 95% CI of the stature exponent encompasses unity, suggesting that a linear function of stature is the appropriate dimension associated with pre‐training REP.

**TABLE 2:** The fitted parameters obtained from the ANCOVA analysis of pre‐training Ln(REP).

Dependent variable: Ln(DistancePre)						
Parameter	*B*	Std. error	*t*	Sig.	95% Confidence interval	
					Lower bound	Upper bound
Intercept Ln(*a*)	1.677	1.286	1.304	0.195	−0.876	4.230
Ln(Stature)	0.769	0.255	3.015	0.003	0.263	1.276
Age (years)	0.063	0.033	1.886	0.062	−0.003	0.129
*R* ^2^ = 0.148 (Adjusted *R* ^2^ = 0.104)						

*Note*: Trained males were used as the baseline measure, estimated as Ln(*a*), where Ln is the natural logarithm, log_*e*_.

Abbreviations: ANCOVA, analysis of covariance; REP, rowing ergometer performance.

### 3.3 Post‐training rowing ergometer performance

The simple power function model *Y* = *a* · *M* ^*b*^ · *ε* predicting the post‐training REP identified a significant body mass exponent found to be 0.237 (SE = 0.056; *P* < 0.001, 95% CI 0.127–0.347; *R* ^2^ = 0.241 [Adjusted *R* ^2^ = 0.209]).

In contrast to the pre‐training analyses, the ‘three dimensional’ allometric ANCOVA analysis of log‐transformed REP (post‐training) identified main effects of both training group (*P* = 0.02) and sex (*P* = 0.037) but not a training‐by‐sex interaction (*P* = 0.096). The ANCOVA analysis also revealed two significant body size covariates, log‐transformed Ln(Mass) with an exponent of 0.606 (SE = 0.115; *P* < 0.001, 95% CI 0.379–0.834) and a significant *negative* Ln(WC) with an exponent −0.747 (SE = 0.200; *P* < 0.001, 95% CI −1.145 to −0.350), plus a significant ‘age’ term (*P* = 0.019) (Table 3).

**TABLE 3:** The fitted parameters obtained from the ANCOVA analysis of post‐training Ln(REP).

Dependent variable: Ln(DistancePost)						
Parameter	*B*	Std. error	*t*	Sig.	95% Confidence interval	
					Lower bound	Upper bound
Intercept Ln(*a*)	1.677	1.286	1.304	0.195	−0.876	4.230
Ln(Mass)	0.606	0.115	5.288	<0.001	0.379	0.834
Ln(WC)	−0.747	0.200	−3.731	<0.001	−1.145	−0.350
Age (years)	0.073	0.031	2.395	0.019	0.013	0.134
*R* ^2^ = 0.396 (Adjusted *R* ^2^ = 0.357)						

*Note*: Trained males were used as the baseline measure, estimated as Ln(*a*), where Ln is the natural logarithm, log_*e*_.

Abbreviations: ANCOVA, analysis of covariance; REP, rowing ergometer performance.

Our findings, predicting Ln(DistancePost) using the ‘three dimensional’ allometric model (Equation 2), identified two body size terms as significant Ln(*M*) and Ln(WC), suggesting that the ideal body shape associated with REP post‐training should be (*M* ^0.606^∙WC^−0.747^) (Table 2). Physiologically, these findings suggest a body mass divided by WC ratio, where the latter WC term is likely to reflect a measure of adiposity. This combination can therefore be interpreted as a proxy for fat‐free mass. Indeed, if we rounded (*M* ^0.606^∙WC^−0.747^) to approximately (*M*/WC)^0.67^ (assuming a common exponent of 2/3), the term can also be roughly interpretated as the cross‐sectional area of for fat‐free mass.

## 4 DISCUSSION

The main findings of the present study are that a three‐dimensional allometric model (incorporating WC in addition to the more commonly utilised stature and body mass dimensions) was appropriate when scaling REP. However, this was only the case following a 6‐week intervention; at baseline the only anthropometric variable that was important for REP was stature. Furthermore, this highlights a further key finding of the present study; that allometric models for scaling are not stable (as previously assumed in studies that tended to use only one body size ‘scaling’ variable, invariably body mass), but rather, they differ over time, in the case of the present study in response to a 6‐week training intervention. These findings question the previous assumption that allometric scaling models are stable over time.

Previous research has reported on the importance of body mass in determining rowing performance (Nevill et al., 2010; Walters et al., 2023). The initial results from the current study, using the simple power function model, *Y* = *a* · *M* ^*b*^ · *ε*, confirm that having greater a body mass does indeed benefit REP both pre‐ and post‐training. The scaling exponents pre‐training were found to be 0.151 (SE = 0.055; *P* = 0.007, 95% CI 0.041–0.260) and 0.237 (SE = 0.056; *P* < 0.001, 95% CI 0.127–0.347) post‐training. These scaling exponents are not dissimilar to those reported by Nevill et al. (2010) when analysing junior elite male rowers. These authors reported that by simply dividing the Concept II rowing ergometer speed by an individual's body mass (m^0.23^), and generating a ‘power‐to‐weight’ ratio (ergometer speed × m^−0.23^), Concept II rowing performance then better reflected actual rowing performance on water. Furthermore, when including body mass as a covariate in analyses examining the efficacy of a rowing intervention involving the participants from the current study, it was noted that heavier adolescents rowed farther, and that for every kg increase in body mass, the distance rowed increased by 1.3 m pre‐ and 1.9 m post‐6‐weeks of training (Walters et al., 2023).

However, the findings of the present study suggest that these simple power function models are too simplistic. By adopting the more flexible three‐dimensional allometric models (Equations 1 and 2), new insights were obtained. The pre‐training ANCOVA identified only one significant body size covariate when predicting pre‐training Ln(REP), that being log‐transformed stature (Ln(HT)) (the stature exponent being 0.769), although ‘age’ was close to significance (*P* = 0.062) (Table 2). The body mass and WC terms were found to be redundant for predicting pre‐training Ln(REP) during the backward elimination process. Clearly, being taller appears to be the most important body shape characteristic associated with superior pre‐trained REP, as opposed to being heavier (greater mass) as assumed if the simple power function model described earlier had been adopted.

In marked contrast, the post‐training ANCOVA identified two significant body size covariates when predicting post‐training Ln(REP), those being a positive Ln(Mass) (exponent was 0.606; SE = 0.115; *P* < 0.001) and a negative Ln(WC) (exponent was −0.747; SE = 0.200; *P* < 0.001), together with a significant ‘age’ term (Table 3). After 6‐weeks of training, being taller was no longer a key predictor of REP; but being older, heavier, and having a smaller WC were. The combination of being heavier with a smaller WC suggests that a key anthropometric characteristic for REP after 6‐weeks of training is a greater lean body mass.

A further key finding of the present study was the differences in the optimal allometric model when comparing pre‐ and post‐training REP. This is an important finding as it questions the assumption in previous studies that scaling models remain stable over time. Indeed, our findings suggest that following a 6‐week period of training the key anthropometric variables changed from stature (pre‐training) to body mass and WC (post‐training). The findings of the present study thus recommend that scaling models should be performed separately when examining performance variables over time, for example, either as part of the ageing process, or in response to an intervention (in the case of the present study, a 6‐week exercise training intervention). Indeed, even a relatively modest 6‐week exercise training intervention in the present study demonstrated the instability of scaling models over time; a key consideration for future research when applying allometric models to children and young people.

Whilst providing novel insights regarding three‐dimensional allometric scaling, and its stability over time, the present study is not without limitations. The sample size in the present analysis can be seen as a potential limiting factor. Scaling is more often performed using a greater number of participants, and future research should examine the changes in scaling models over time in larger samples. Future research should also aim to examine whether maturation is a key predictor in scaling models such as those examined in the current study; and could consider using a multilevel modelling approach to further explore the relationship between REP and body size over time.

In summary, the findings of the present study demonstrate the utility of the more flexible and sensitive three‐dimensional allometric model for examining performance differences between individuals of varying body size and composition. In addition, the findings of the present study question the assumption that key body size ‘scaling’ parameters remain stable when assessed over time. Specifically, after 6‐weeks of training, the key predictors of REP in adolescents were a higher body mass, a smaller WC, and being older, whereas in pre‐training, taller stature was the key predictor for superior REP. In conclusion, the findings from the current study (using a novel three‐dimensional allometry approach) emphasise that relatively subtle changes in individuals' behavioural characteristics, such as changes in their training/fitness status, might result in quite dramatic changes in the body dimension characteristics and scaling parameters deemed to be key for performance in activities such as REP.

## CONFLICT OF INTEREST STATEMENT

The underpinning original research study was funded by Concept2 Ltd. The authors declare that the research was conducted in the absence of any commercial or financial relationships that could be construed as a potential conflict of interest.

## Data Availability

Data are available from corresponding authors upon reasonable request.
